# FACTORS ASSOCIATED WITH LIFE SATISFACTION IN VETERANS IN THE HEALTHY AGING PROJECT: BRAIN PSYCHOEDUCATIONAL CLASS

**DOI:** 10.1093/geroni/igae098.3397

**Published:** 2024-12-31

**Authors:** Maulika Kohli, Alice Verstaen, Emily Trittschuh

**Affiliations:** VA Puget Sound Healthcare System, Seattle, Washington, United States; Lyra Health, Burlingame, California, United States; VA Puget Sound Healthcare System, Seattle, Washington, United States

## Abstract

The Healthy Aging Project-Brain (HAP-B) was developed to encourage engagement in activities associated with successful aging. Across 6 sessions, HAP-B targets sleep, socialization, physical, and cognitive activity through myth-busting, developing SMART goals, and tracking behavioral change. This study aims to examine associations between physical and mental health factors with life satisfaction. Veteran participants (n=50, mean age = 70.6 years) were predominantly male-identity (88%) and White (76%). Linear regressions examined associations between physical and emotional functioning factors and life satisfaction at baseline, covarying for age. Results indicate that higher life satisfaction is significantly associated with lower depressive symptoms, higher emotional well-being, and higher self-efficacy in our sample. HAP-B is effective in facilitating behavioral change that has downstream positive effects on emotional functioning. It is feasible, accessible, and scalable, supporting a model of care among older adult Veterans that combines psychoeducational and skill-based approaches. Future groups may emphasize emotional well-being as a direct intervention target. There is a significant need to prioritize engagement with, and accessibility of, mental healthcare services to improve both emotional well-being and overall satisfaction with life. Healthcare providers are well-positioned to encourage engagement with activities that are protective against adverse cognitive aging.

